# Lysosomal targeting of liposomes with acidic pH and Cathepsin B induces protein aggregate clearance

**DOI:** 10.1186/s12964-025-02310-z

**Published:** 2025-06-19

**Authors:** Minsol Jeon, Da-Eun Kim, So Young Choi, Seoyoung Kim, Seongchan Kim, Hyojin Lee, Hyunkyung Kim

**Affiliations:** 1 Department of Biochemistry and Molecular Biology, Korea University College of Medicine, Seoul, 02841 Republic of Korea; 2 BK21 Graduate Program, Department of Biomedical Sciences, Korea University College of Medicine, Seoul, 02841 Republic of Korea; 3 Biomaterials Research Center, Biomedical Research Division, Korea Institute of Science and Technology (KIST), Seoul, 02792 Republic of Korea; 4 School of Biomedical Engineering, College of Health Science, Korea University, Seoul, 02841 Republic of Korea; 5 College of Pharmacy and Research Institute of Pharmaceutical Sciences, Gyeongsang National University, Jinju, 52828 Gyeongsangnam-do Republic of Korea; 6 Division of Bio-Medical Science & Technology, KIST School, Korea University of Science and Technology (UST), Seoul, 02792 Republic of Korea; 7 Department of Integrative Biotechnology, College of Biotechnology and Bioengineering, SKKU-KIST, Sungkyunkwan University, Suwon, 16419 Gyenggi Republic of Korea

**Keywords:** Proteinopathy, Lysosome, Aggregate clearance, Cathepsin, Autophagy

## Abstract

The autophagy-lysosomal pathway is a cellular degradation mechanism that regulates protein quality by eliminating aggregates and maintaining normal protein function. It has been reported that aging itself reduces lysosomal proteolytic activity in age-related neurodegenerative disorders such as Alzheimer’s disease and Parkinson’s disease. Reduction in lysosomal function may underlie the accumulation of protein aggregates such as amyloid beta (Aβ), tau, and α-synuclein. Some of these protein aggregates may cause additional lysosomal dysfunction and create a vicious cycle leading to a gradual increase in protein aggregation. In this study, liposome-based lysosomal pH-modulating particles (LPPs), containing a liquid solution to adjust lysosomal pH, have been developed to restore lysosomal function. The results demonstrate that acidic LPPs effectively restore lysosomal function by recovering lysosomal pH and facilitating the removal of protein aggregates. These findings demonstrated that acidic LPPs could effectively recover the abnormal lysosomal function via restoration of lysosomal pH and enhance the clearance of protein aggregates. Furthermore, the simultaneous introduction of Cathepsin B (CTSB) proteins and acidic LPP revealed a synergistic effect, promoting lysosomal pH recovery and enhancing aggregates removal. These findings suggest a novel strategy for improving lysosomal clearance activity in proteinopathies.

**Supplementary Information:**

The online version contains supplementary material available at 10.1186/s12964-025-02310-z.

## Introduction

Proteinopathy, also known as protein conformational disorder, is a prominent feature in which protein folding failure can lead to cytotoxicity and loss of biological function. The development of various neurodegenerative diseases is closely associated with proteinopathy [1–3]. Misfolded or structurally altered proteins can self-assemble into aggregates and continuously generate insoluble deposits, ultimately leading to proteotoxicity and disease progression [4–7].

Protein aggregation refers to the process by which proteins misfold and accumulate into insoluble fibrils or plaques within the tissue, disrupting cellular function and contributing to cellular toxicity [8, 9]. Essential proteins, such as amyloid beta (Aβ) and tau in Alzheimer’s disease (AD), α-synuclein in Parkinson’s disease (PD), and huntingtin in Huntington’s disease (HD), have been identified as major players in this process. Under pathological conditions, these proteins undergo conformational changes that promote their aggregation into oligomers and fibrils, which are toxic to neurons. The presence of these aggregates is strongly correlated with disease progression, highlighting their role as biomarkers of disease and potential targets for intervention [10–15].

Numerous studies have focused on identifying key factors that cause protein aggregation and influence the rate of degeneration. However, five out of seven FDA-approved treatments for AD primarily serve to relieve symptoms. As a result, there is increasing interest in developing treatments that target more fundamental causes, offering solutions that directly address and remove protein aggregates [16, 17].

Lysosomes, which are involved in macroautophagy, chaperone-mediated autophagy, and endosomal microautophagy, serve as organelles responsible for protein degradation. Autophagy, in particular, represents the primary mechanism for protein aggregate clearance and involves the sequestration of substances within autophagosomes, their subsequent fusion with lysosomes, and their degradation by various hydrolytic enzymes within the lysosomal environment [18–20].

It has been established that protein aggregation inhibits autophagy and lysosomal activity [21–27]. However, our current investigation provides novel evidence that different types of protein aggregation directly influence lysosome functionality, specifically by impeding lysosomal acidification and cathepsin activity. This finding reveals previously unexplored aspects of proteinopathies and their impact on cellular degradation mechanisms, highlighting the importance of lysosomal dysregulation in the context of disease progression.

The treatment of brain diseases, including AD, is often hindered by the restrictive nature of blood‒brain barrier (BBB). It has been reported that lipid-based nanoparticles enhance the delivery of therapeutic agent across the BBB [28–32]. Among these, liposomes are biocompatible and biodegradable carriers that offer improved stability and reduced toxicity [33–36]. In this study, we developed liposome-based lysosomal pH-modulating particles (LPPs) by encapsulating acidic pH solutions, and further engineered CTSB-loaded LPPs (CTSB-LPPs) by incorporating CTSB proteins. The efficacy of these constructs in the degradation of protein aggregates was evaluated. The findings of this study demonstrate that both acidic LPPs and CTSB-LPPs have the capacity to effectively break down protein aggregates. This suggests that liposome-based acidic LPPs have the potential to be used as a promising therapeutic platform for treating proteinopathies in the brain.

## Results

### Protein aggregates lead to defects in lysosomal acidification and decreased cathepsin activity

**Fig. 1:**
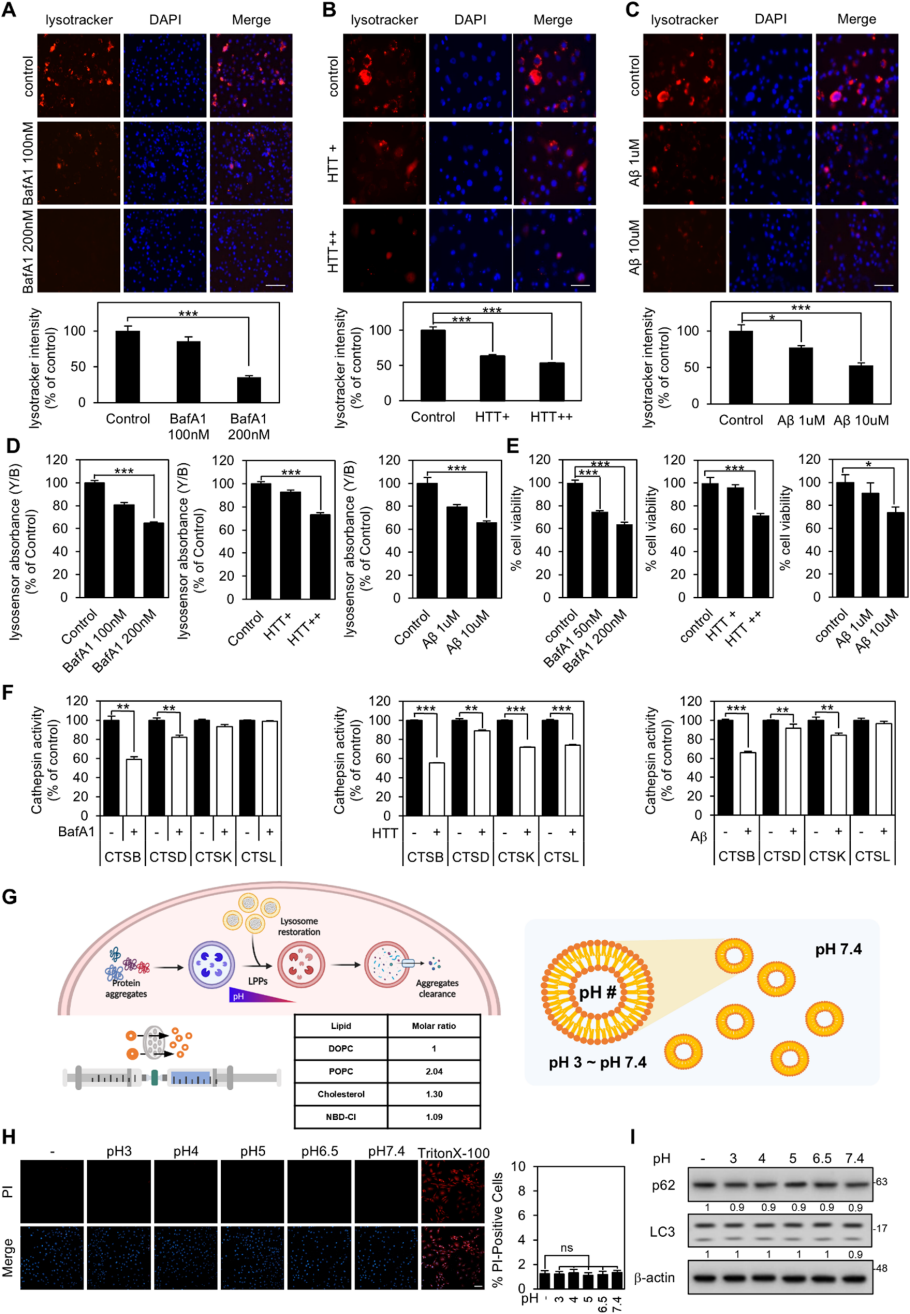
Protein aggregates led to defects in lysosomal acidification and decreased cathepsin activity. (**A**) Lysosomal acidification analysis in U87MG cells following 8 h treatment with BafA1 at 100–200 nM using LysoTracker staining, with quantification per field (*n* = 5 biological replicates). Scale bar represents 275 μm. (**B**, **C**) LysoTracker activity was analyzed in U87MG and SK-N-SH cells after overexpression of HTT Q74 exon 1 (0.5–1 µg) and treatment with Aβ oligomers (1–10 µM), with quantification performed per field (*n* = 5 biological replicates). Scale bar represents 125 μm. The fluorescence intensity was quantified and normalized to the control using ImageJ software. (**D**) LysoSensor Yellow/Blue absorbance analysis in U87MG and SK-N-SH cells under the same experimental conditions as in (**A**, **B**, **C**) (*n* = 5 biological replicates). (**E**) Cell viability was measured using MTS assay 24 h after treatment (*n* = 8 biological replicates). Data are presented as bar graphs showing mean ± S.E. The *p*-values were calculated using one-way ANOVA with Bonferroni correction (*, *p* < 0.05, **, *p* < 0.01; ***, *p* < 0.001). (**F**) Cathepsin activity assay in U87MG and SK-N-SH cell lines after 200 nM BafA1 treatment, overexpression of HTT Q74 exon 1 (1 µg), and treatment of Aβ oligomers (10µM). Cathepsin activity was quantified and normalized to the control (*n* = 3 biological replicates). Statistical significance was determined using a t-test (*, *p* < 0.05; **, *p* < 0.01; ***, *p* < 0.001). (**G**) Schematic illustration depicting the function of LPPs and the synthesis of LPPs with different pH. (**H**) Cell death analysis after 100 µg/mL LPPs treatment assessed by PI staining and quantified per field (*n* = 5 biological replicates). Scale bar represents 40 μm. (**I**) Western blot analysis of autophagy flux markers after 100 µg/mL LPPs treatment for 24 h. The– indicates non-treated control. Band intensities were analyzed using ImageJ and normalized to actin as a loading control. Relative intensity values are shown below each band

The clearance of protein aggregates is closely linked to the function of lysosomes, which are crucial organelles responsible for their degradation. Understanding lysosomal dysfunction in protein aggregation is critical since impaired lysosomal function can contribute to disease progression by exacerbating protein aggregation or impeding efficient clearance processes [37–39]. To accurately assess the impact of protein aggregates on lysosomal pH accurately, we utilized LysoTracker staining. This technique selectively labels acidic organelles in live cells to monitor changes in lysosomal acidification. Disruption of lysosomal acidification was achieved via the use of bafilomycin A1 (BafA1), an inhibitor of vacuolar H^+^ ATPase (V-ATPase), which increases lysosomal pH. After 8 h of BafA1 treatment, the number of acidic lysosomes decreased in a concentration-dependent manner (Fig. 1A). Moreover, a similar correlation was observed in cells overexpressing the polyglutamine form of Huntingtin (HTT Q74 exon 1), an essential protein involved in Huntington’s disease, and in cells treated with Aβ oligomers (Fig. 1B and C). To confirm these observations, we employed LysoSensor Yellow/Blue, a ratiometric dye that allows for precise measurement of lysosomal pH changes based on its fluorescence emission shift under varying pH conditions. In agreement with LysoTracker results, LysoSensor Yellow/Blue revealed a concentration-dependent increase in lysosomal pH following BafA1 treatment (Fig. 1D). Similarly, in cells overexpressing HTT Q74 exon 1 or exposed to Aβ oligomers, the fluorescence ratio indicative of lysosomal acidity was significantly altered, reflecting reduced lysosomal acidification (Fig. 1D). On the basis of these findings, we propose that protein aggregates interfere with lysosomal acidification. Furthermore, we found that protein aggregation led to impaired lysosomal acidification, resulting in decreased cell viability (Fig. 1E).

Cathepsin enzymes inside lysosomes play important roles in the degradation of various biomolecules, including proteins, nucleic acids, lipids, and carbohydrates [40, 41]. Lysosomal dysfunction or altered pH conditions may result in decreased cathepsin activity, which may have a deleterious effect on cellular degradation processes. To determine the effects of different types of protein aggregates on cathepsin enzyme activities within lysosomes and their associations with lysosomal acidification impairment, we examined the activity of four cathepsin enzymes: Cathepsin B (CTSB), Cathepsin D (CTSD), Cathepsin K (CTSK), and Cathepsin L (CTSL). In response to treatment with BafA1, CTSB activity was significantly lower than that of other cathepsin enzymes. Furthermore, the overexpression of HTT Q74 exon 1 led to decreased CTSK and CTSL activities, while CTSB exhibited the most notable reduction. Similarly, treatment with Aβ oligomers resulted in a significant decrease in CTSB activity and a weak reduction in CTSK activity (Fig. 1F). These findings underscore the harmful impact of inhibiting lysosomal acidification on the activity of cathepsin enzymes, with CTSB showing the most significant decline, particularly in the presence of protein aggregates. Therefore, restoring lysosomal acidification and enhancing the function of CTSB enzymes are crucial for alleviating adverse effects on cells and facilitating the clearance of protein aggregates. To this end, we designed and applied liposome-based lysosomal pH-modulating particles (LPPs).

For the synthesis of LPPs, we used 1,2-dioleoyl-sn-glycero-3-phosphocholine (DOPC), 1-palmitoyl-2-oleoyl-sn-glycero-3-phosphocholine (POPC), cholesterol, and 1-palmitoyl-2-{12-[(7-nitro-2-1,3-benzoxadiazol-4-yl)amino]dodecanoyl}-sn-glycero-3-phosphocholine (16:0–12:0 NBD-PC), selected based on their known interactions with lysosomes (Fig. 1G). POPC and DOPC are both phosphatidylcholine (PC) lipids that differ in acyl chain length and degree of unsaturation. DOPC (18:1 PC) contains two 18-carbon unsaturated acyl chains and functions as a neutral helper lipid that promotes the formation of stable LPPs [42]. POPC (16:0–18:1 PC) contains one saturated 16-carbon (palmitoyl) chain and one unsaturated 18-carbon (oleoyl) chain, and is widely used to mimic eukaryotic cell membranes and enhance structural integrity and cellular uptake [43]. 16:0–12:0 NBD-PC is a phosphatidylcholine derivative consisting of a 16-carbon acyl chain and a 12-carbon acyl chain conjugated with the fluorescent dye 12-[(7-nitro-2-1,3-benzoxadiazol-4-yl)amino]dodecanoyl (NBD). NBD is commonly used to label and track cellular lipids, with excitation/emission peaks at 464/531 nm.

Cholesterol, a critical regulator of endocytosis and a precursor for steroid hormones and vitamins, was included to enhance membrane rigidity and stability [44–46]. According to previous studies, lysosomal integral membrane protein 2 (LIMP-2/SCARB2), interacts extensively with various phosphatidylcholine (PC) lipids and cholesterol, thereby modulating its own functional activity [47]. Based on these findings, we incorporated PC lipids and cholesterol into the LPP formulation to promote lysosomal integration. LPPs are designed to either burst or accumulate within lysosomes following cellular uptake. The preparation and characterization of LPPs, including dynamic light scattering (DLS) with polydispersity index (PDI) and cryo-transmission electron microscopy (cryo-TEM), are detailed in Tables 1 and 2 and Fig. S1A–C.

The endosome–lysosome pathway is frequently considered a significant impediment in nanocarrier-mediated drug delivery due to lysosomal degradation of therapeutic agents. In contrast, our approach takes advantage of this intrinsic intracellular trafficking route. Nanocarriers are typically internalized into endocytic vesicles, fuse with early endosomes (EE), mature into late endosomes (LE), and are ultimately trafficked to lysosomes [48]. Rather than avoiding lysosomal degradation, we engineered lysosome-targeting LPPs that leverage this natural pathway. The specificity of lysosomal targeting was confirmed by co-localization analysis using Cy7-labeled LPPs and lysosome-associated membrane protein 2 (LAMP2) staining (Fig. S1D, S1E). Our results showed that the LPPs did not induce cytotoxicity, affect cell proliferation, or cause cell death, as assessed via lactate dehydrogenase (LDH) assays, MTS assays, and propidium iodide (PI) staining (Fig. S1F, S1G, 1H). Furthermore, the expression of representative autophagy markers, including a reduction in p62 expression and LC3 lipidation, was not directly altered by LPP treatment alone in the absence of induced protein aggregates (Fig. 1I).

**Table 1:** The sizes of LPPs with different pH values

pH	Size (d.nm)
pH 3	116.54
pH 4	117.73
pH 5	123.3
pH 6.5	135.33
pH 7.4	111.26

**Table 2:** Zeta potential of LPPs at different pH values

pH	Zeta Potential	Stedv (mV)
	(mV)	
pH 3	-1.71	1.25
pH 4	0.158	0.259
pH 5	-0.287	0.406
pH 6.5	-0.357	0.225
pH 7.4	0.018	0.268

### Acidic LPPs restore lysosomal function and CTSB activity

**Fig. 2:**
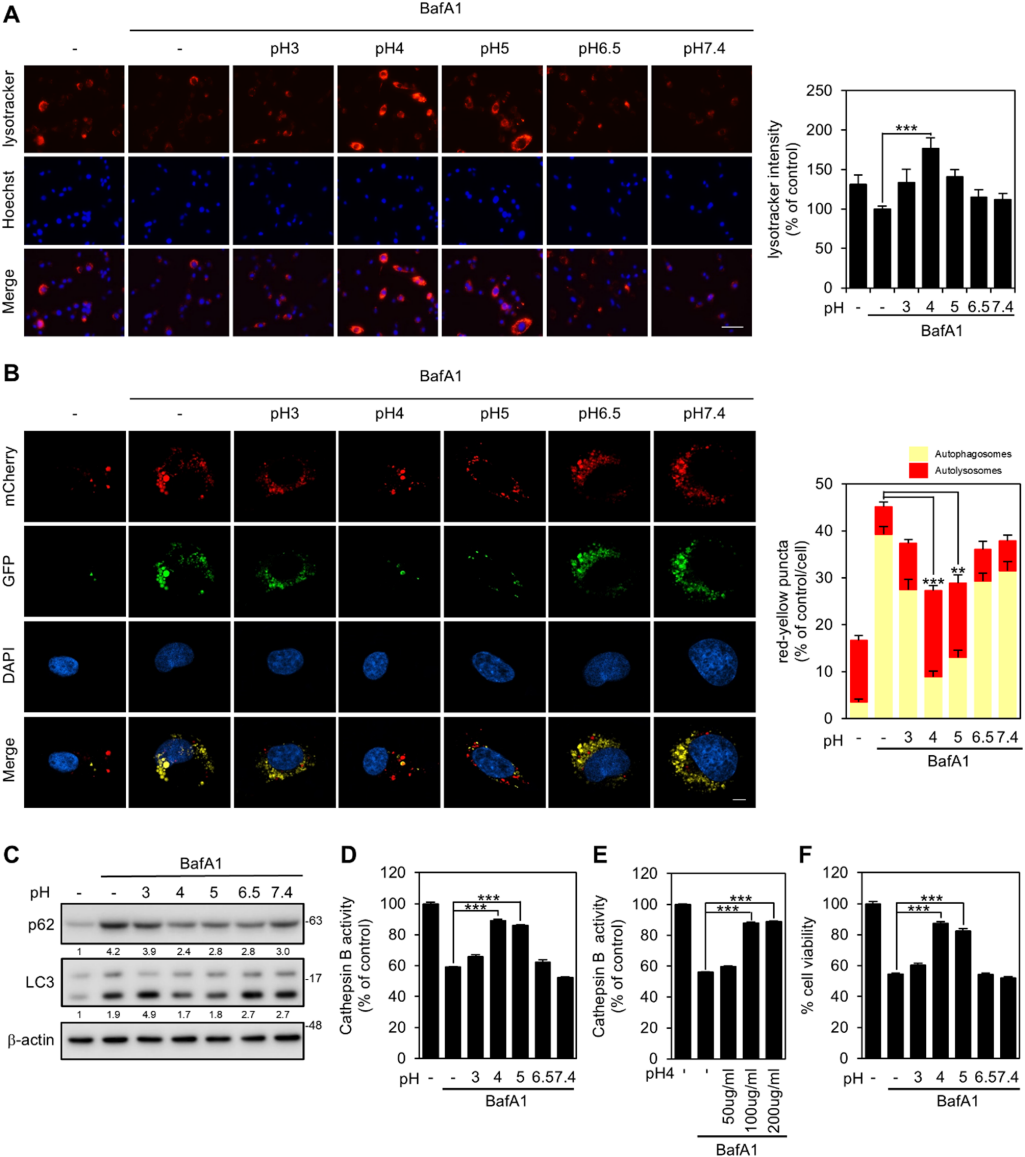
Acidic LPPs restored lysosomal acidification and CTSB activity. (**A**) Analysis of LysoTracker staining intensity in U87MG cells after treatment with 200 nM BafA1 and 100 µg/mL LPPs for 6 h. LysoTracker fluorescence intensities were quantified per field and normalized to the control (*n* = 6 biological replicates). Scale bar represents 125 μm. (**B**) The autophagy flux was measured after mCherry-GFP-LC3 transfection in U87MG cells under treatment with 100 µg/mL LPPs of different pH. The ratio of red to yellow puncta was analyzed per cell using ImageJ-based analysis (*n* = 3 biological replicates). Scale bar represents 5 μm. (**C**) Western blot analysis of autophagy flux markers after treatment with 200 nM BafA1 and 100 µg/mL LPPs for 6 h. Protein expression levels of p62 and LC3 were assessed by Western blot. Band intensities were analyzed using ImageJ and normalized to actin as a loading control. Relative intensity values are shown below each band. (**D**, **E**) Cathepsin B activity in U87MG treated with BafA1 (*n* = 4 biological replicates) and different pH LPPs or different concentrations of pH 4 LPPs (*n* = 6 biological replicates) for 6 h. (**F**) Cell viability analysis under 200 nM BafA1 treatment with 100 µg/ml LPPs of different pH (*n* = 10 biological replicates). Data are presented as bar graphs showing mean ± S.E. The *p*-values were calculated using one-way ANOVA with Bonferroni correction (*, *p* < 0.05, **, *p* < 0.01; ***, *p* < 0.001)

To investigate the impact of acidic LPPs on intracellular lysosomal pH changes, we employed LysoTracker staining to label acidic organelles. The results revealed that the pH increase caused by BafA1 was restored to levels exceeding normal conditions when the cells were treated with LPPs at pH 4. The pH 4 LPPs exhibited significantly greater activity than the LysoTracker signal intensity observed in normal cells (Fig. 2A). When lysosomes fuse with autophagosomes to form autolysosomes, fluorescence persists from the acid-stable mCherry component, whereas the fluorescence of the acid-labile GFP component decreases. This enables the visualization of autophagosomes in yellow and autolysosomes in red [49]. In the presence of BafA1, a compound that inhibits the maturation of autophagic vacuoles and thereby prevents lysosome fusion, autophagosome accumulation occurs [50]. However, the inhibitory effects of BafA1 could be reversed by treatment with pH 4 and pH 5 LPPs. Among the tested formulations, pH 4 LPPs most effectively reversed the inhibitory effects of BafA1. (Fig. 2B). These results indicated that acidic LPPs facilitated efficient conversion from autophagosomes to autolysosomes. In addition, we validated the results by examining changes in the expression of autophagy markers, specifically p62 and LC3-II, through western blot analysis. When BafA1 was applied, p62 accumulation and lipidated LC3-II were simultaneously observed, confirming the inhibition of autophagy activity. However, when pH 4 and pH 5 LPPs were administered, there was an increase in autophagy flux following the degradation of both p62 and a decrease in LC3-II (Fig. 2C). Therefore, restoring lysosomal acidification enhances autophagy flux to facilitate the efficient degradation of autophagosomes and their cargo. Furthermore, acidification of lysosomes has been confirmed to restore the activity of CTSB. Treatment with BafA1 resulted in a decrease in CTSB activity, which was most effectively restored to normal levels by pH 4 LPPs (Fig. 2D). This was further confirmed by measuring CTSB activity across different concentrations of pH 4 LPP treatment (Fig. 2E). Additionally, the compromised cell viability caused by BafA1 was reversed upon treatment with pH 4 and pH 5 LPPs (Fig. 2F). These results suggest that acidic LPPs facilitate the functional recovery of lysosomes, promote autophagy, and prevent cell death.

### Acidification of lysosomes through LPPs restores lysosomal dysfunction caused by protein aggregates

**Fig. 3:**
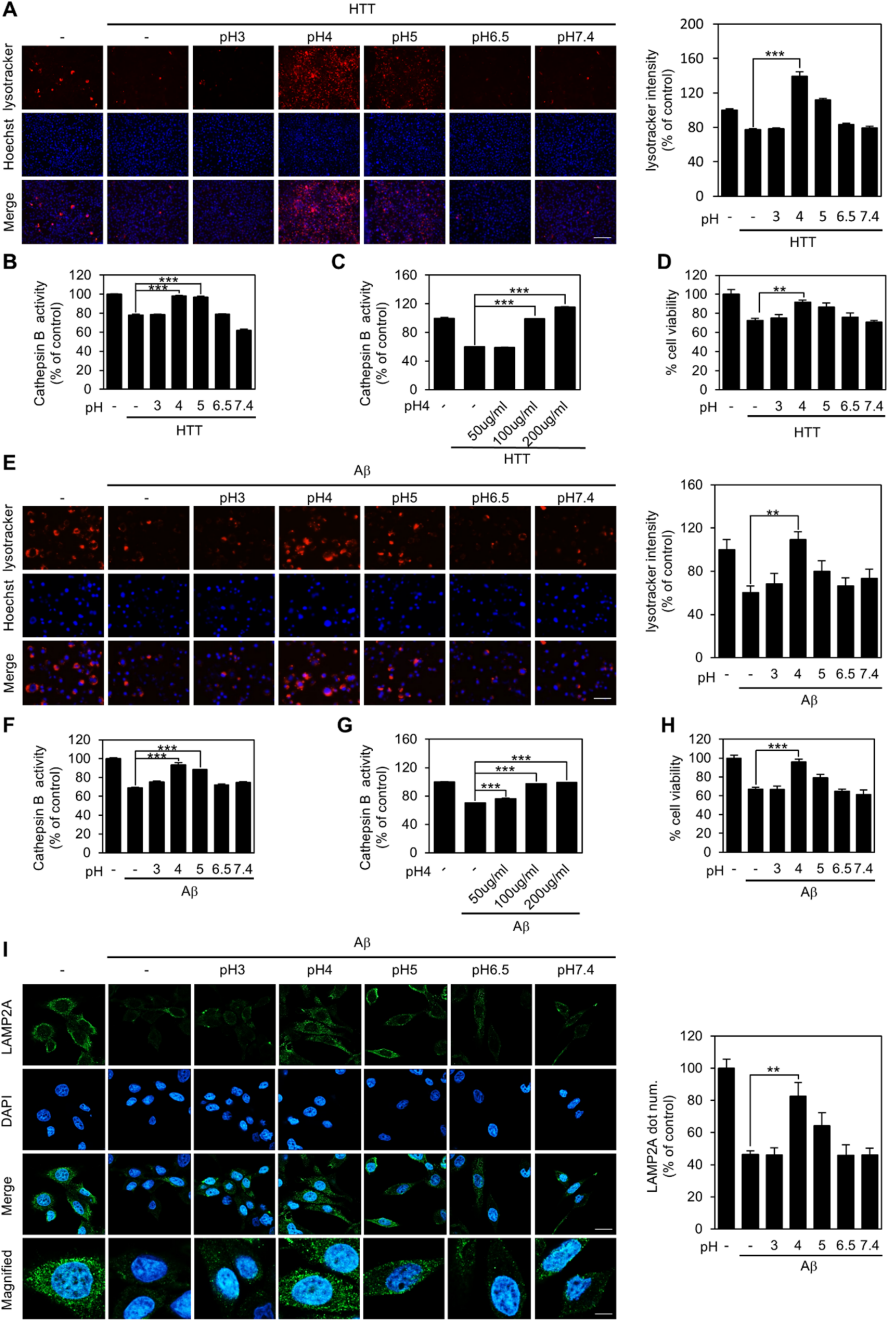
Acidification of lysosomes through LPPs restored lysosomal dysfunction caused by protein aggregates. (**A**) LysoTracker staining intensity in U87MG cells after 24-hour transfection with HTT Q74 exon 1 (1 µg) and 6-hour treatment with 100 µg/ml LPPs of different pH was quantified per field (*n* = 5 biological replicates). Scale bar represents 275 μm. (**B**) CTSB activity was analyzed under identical conditions of (**A**) (*n* = 4 biological replicates). (**C**) CTSB activity was analyzed at various concentrations of pH 4 LPPs (50–200 µg/ml) (*n* = 6 biological replicates) for 6 h. (**D**) Cell viability analysis under HTT Q74 exon 1 transfection with 100 µg/ml LPPs of different pH (*n* = 10 biological replicates). (**E**) LysoTracker activity in SK-N-SH cells after 24-hour treatment with 10 µM Aβ oligomers followed by 6-hour treatment with 100 µg/ml LPPs of different pH was quantified per field (*n* = 5 biological replicates). Scale bar represents 125 μm. (**F**, **G**) CTSB activity was analyzed under identical conditions of (**E**) (*n* = 4 biological replicates) and different concentration of pH 4 LPPs (50–200 µg/ml) (*n* = 6 biological replicates) for 6 h. (**H**) Cell viability analysis under Aβ oligomers treatment with 100 µg/ml LPPs of different pH (*n* = 10 biological replicates). (**I**) SK-N-SH cells were treated with 10 µM of Aβ oligomers for 24 h and visualized using LAMP2A antibody, and quantified per cell (*n* = 5 biological replicates). Scale bars: 10 μm (overview), 3 μm (magnified). Data are presented as bar graphs showing mean ± S.E. The *p*-values were calculated using one-way ANOVA with Bonferroni correction (*, *p* < 0.05, **, *p* < 0.01; ***, *p* < 0.001)

We next assessed whether acidic LPPs could restore lysosomal acidification impaired by protein aggregation, using LysoTracker and LysoSensor staining. The overexpression of HTT Q74 exon 1 and the application of Aβ oligomers resulted in reduced lysosomal acidification, which was most effectively ameliorated by pH 4 LPPs (Fig. 3A and E, S2A). Notably, lysosomal pH measurements revealed that the Aβ-induced increase in lysosomal pH was mitigated to a normal range by treatment with pH 4 and 5 LPPs (Fig. S2E). The reduction in CTSB activity caused by HTT Q74 exon 1 overexpression and Aβ oligomer treatment was nearly restored to normal levels by pH 4 LPPs treatment under both lethal and non-lethal conditions (Fig. 3B and F, S2B, S2C). In addition, we found that pH 4 LPPs, which were the most effective in degrading aggregated proteins, enhanced CTSB activity and promoted protein degradation in a concentration-dependent manner (Fig. 3C and G). Furthermore, treatment with pH 4 LPPs alleviated the reduction in cell viability induced by protein aggregation under lethal conditions. (Fig. 3D and H).

Specifically, it has been reported that Aβ binds to the lysosomal membrane, increasing lysosomal membrane permeability. This results in the leakage of proteases from lysosomes into the cytoplasm, leading to impaired lysosomal function and destabilization of LAMP2, a membrane protein abundantly expressed in lysosomes, which consequently undergoes degradation [51, 52]. Previous studies have also shown that high concentrations of Aβ oligomers impair autophagy and lysosomal function in RPE cells, accompanied by reduced LAMP2 expression [53, 54]. Therefore, we investigated whether protein aggregates affect LAMP2 expression and whether acidic LPPs could restore it. The introduction of Aβ oligomers resulted in a decrease in LAMP2 expression. However, following treatment with pH 4 LPPs, the restoration of LAMP2 expression was confirmed through immunocytochemistry (Fig. 3I).

### Restoring lysosomal acidification promotes the clearance of protein aggregates and the combined treatment of CTSB-LPPs facilitates the removal of protein aggregates

**Fig. 4:**
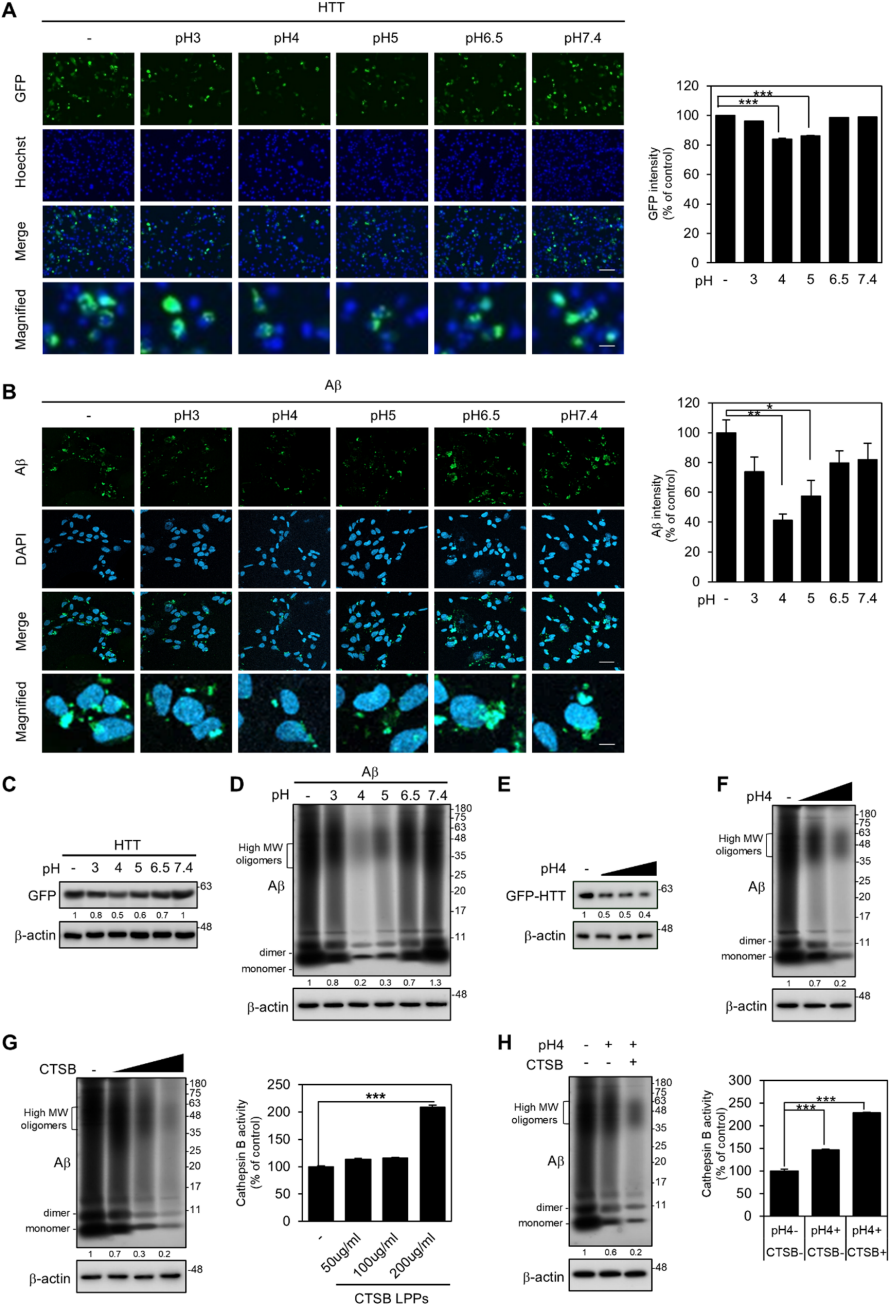
Restoring lysosomal acidification promoted the clearance of protein aggregates and the combined treatment of LPPs facilitated the removal of protein aggregates. (**A**) GFP intensity was measured per field after GFP-HTT Q74 exon 1 overexpression and treatment with various pH LPPs (100 µg/ml) for 72 h in U87MG cells (*n* = 3 biological replicates). Scale bars: 275 μm (overview), 55 μm (magnified). (**B**) Aβ oligomers were stained and measured per field after treatment with 10 µM Aβ oligomers with various pH LPPs (100 µg/ml) for 72 h in SK-N-SH cells (*n* = 5 biological replicates). Scale bars: 20 μm (overview), 4 μm (magnified). (**C**, **D**) Western blot analysis of protein expression of HTT Q74 exon 1 and Aβ oligomers after LPPs treatment. (**E**, **F**) Western blot analysis of protein expression of HTT and Aβ after treatment of pH 4 LPPs in a dose-dependent manner (50–200 µg/ml). (**G**) Western blot analysis of protein expression of Aβ oligomers and Cathepsin B activity after treatment with CTSB-LPPs (50–200 µg/ml) for 24 and 6 h, respectively (*n* = 6 biological replicates). (**H**) Western blot analysis of Aβ oligomers and Cathepsin B activity after combined treatment with pH 4 LPPs (100 µg/mL) and CTSB-LPPs (100 µg/mL) for 24 and 6 h, respectively (*n* = 6 biological replicates). Band intensities were analyzed using ImageJ and normalized to actin as a loading control. Relative intensity values are shown below each band. Data are presented as bar graphs showing mean ± S.E. The *p*-values were calculated using one-way ANOVA with Bonferroni correction (*, *p* < 0.05, **, *p* < 0.01; ***, *p* < 0.001)

To investigate the effects of acidic LPPs on protein aggregate degradation through lysosomal acidification and increased CTSB activity, we performed immunocytochemistry to measure the fluorescence intensity of protein aggregates. The expression of both HTT Q74 exon 1 and Aβ oligomers significantly decreased upon delivery of pH 4 LPPs, accompanied by colocalization between LAMP2 and aggregates (Fig. 4A and B, S3A, S3B). This result was further supported by the decrease in protein levels observed via Western blot analysis (Fig. 4C and D). In addition, pH 4 LPPs promoted the decomposition of HTT Q74 exon 1 and Aβ oligomers in a concentration-dependent manner (Fig. 4E and F).

Additionally, we employed the PROTEOSTAT^®^ Aggresome Detection Reagent, a quantitative measurement method that labels protein aggregates without introducing protein mutations or oligomers. We induced proteasome dysfunction via the proteasome inhibitor MG132. This treatment forms an aggresome to sequester misfolded proteins and reduce proteotoxic stress. Aggresomes can be degraded by autophagy as a compensatory mechanism for maintaining protein homeostasis. We found that pH 4 LPPs most effectively inhibited aggresome formation in the environment where protein aggregation was induced by MG132. (Fig. S2D). In conclusion, our findings indicate that acidic LPPs enhance lysosomal function, leading to the degradation of protein aggregates.

The activity of CTSB was primarily impaired by BafA1 treatment or protein aggregation. To address this, we designed and developed LPPs encapsulating recombinant CTSB proteins, referred to as CTSB-LPPs. To develop a more effective method to eliminate protein aggregation while restoring lysosomal acidification, we conducted simultaneous treatment and observation using pH 4 and CTSB-LPPs. To ensure a suitable pH environment for CTSB activity during the experiment, we loaded the CTSB protein in pH 5 LPPs for testing. The application of CTSB at pH 5 led to a concentration-dependent reduction in Aβ oligomers level (Fig. 4G). In addition, simultaneous treatment of CTSB-LPPs with pH 4 LPPs resulted in more effective degradation of Aβ oligomers than treatment with pH 4 LPPs alone (Fig. 4H). In conclusion, our findings indicate that the combined approach of introducing the CTSB protein and restoring lysosomal acidification exhibited a strong synergistic effect on enhancing lysosomal function. This synergistic effect is crucial for achieving more comprehensive recovery and improving the clearance of protein aggregates.

## Discussion

Our results are consistent with those of previous studies that emphasized the important role of the optimal lysosomal pH in protein degradation, demonstrating that restoring lysosomal acidification can significantly improve lysosomal function impaired by protein aggregation. Our study provides direct evidence that precise regulation of lysosomal pH, when protein aggregation is induced, can significantly reduce the burden of protein aggregation, suggesting a potential therapeutic approach for diseases caused by protein aggregation.

In addition to its effect on lysosomal pH, protein aggregation was found to inhibit the function of cathepsin enzymes, the major hydrolases operating within the lysosome. Notably, although CTSB function was commonly reduced, the other cathepsin types affected by protein aggregation varied. Our study revealed that cells treated with pH 4 LPPs recovered the function of the lysosomal hydrolase CTSB, which degrades aggregated proteins more efficiently. Furthermore, we observed a synergistic effect when pH 4 LPPs and CTSB-LPPs were added. This finding suggests a more effective treatment method by enhancing enzyme function in lysosomes through pH recovery and active delivery to CTSB, which is most affected by protein aggregation. It is important to note that lysosomes naturally function optimally in an acidic environment of approximately pH 4.5–5.0. When exposed to excessively low pH, the stability of lysosomal membrane proteins and phospholipids may be compromised, potentially leading to lysosomal membrane damage [55]. Lysosomes contain over 50 different hydrolases, most of which function optimally at pH 4.6–5.0 [56]. Specifically, Cathepsin B, a major lysosomal hydrolase, exhibits peak activity around pH 4.6 [57]. Therefore, extremely low pH, such as pH 3, can impair the enzymatic activity of Cathepsin B and other lysosomal hydrolases, rather than enhancing them.

In the future, it will be possible to tailor delivery to form more diverse combinations of cathepsins that require recovery for each protein aggregation. Additionally, by supplying intracellular membrane structures, liposome-based delivery can address deficiencies and disruptions in membrane recycling caused by protein aggregates, which are major contributors to the inhibition of the autophagy–lysosomal pathway.

Given the central role of protein aggregation in the pathogenesis of neurodegenerative diseases such as Alzheimer’s, Parkinson’s, and Huntington’s diseases [10–15], our findings have significant therapeutic implications. By enhancing lysosomal function through acidification and restoring dysfunctional endogenous enzymes, we may offer a novel strategy to alleviate the proteotoxic stress observed in these diseases and reduce overload of the protein quality control system. Previous studies have explored and proposed the use of chaperone proteins, inducers of the proteasome and autophagy pathways, small molecules that inhibit protein aggregation, or antibodies that target aggregated proteins [58–63]. Our approach may provide a novel dual strategy to manage disease progression by complementing existing therapies aimed at reducing aggregate formation.

The following limitations and future directions should be considered. In this study, we utilized the Wisniewski (2011) method to induce amyloid-beta oligomerization. However, recent studies have demonstrated that, while both methods remove the β-sheet structure using HFIP, the Berntsson (2023) method further eliminates residual β-sheet structures using NaOH and separates monomers via size-exclusion chromatography. This approach more precisely mimics oligomer formation under physiological conditions (pH 7.4, 37 °C) [64, 65] Given this difference, further validation may be necessary based on more precisely formed Aβ oligomers. The LPPs used to enhance lysosomal acidification may have potential off-target effects. To ensure the specificity and safety of LPPs, the potential risks of chronic treatment must be investigated. In addition, we may consider comparing or combining our approach with existing methods known to dissolve aggregates to achieve effective clearance. Investigating the combined effects of lysosomal acidification with other therapeutic strategies, such as autophagy inducers or approved neurodegenerative disease treatments, will provide a more comprehensive consideration in managing protein aggregation disorders. Furthermore, exploring the role of lysosomal acidification in different cell types and tissues will be critical to understanding the broader applicability of this therapeutic strategy.

## Materials and methods

### Cell culture

Human glioma U87MG cells (KCLB No.30014) and Human neuroblastoma SK-N-SH cells (KCLB No.22266) were purchased from Korean Cell Line Bank (Seoul, Korea) and were cultured in a humidified atmosphere containing 5% CO2 at 37 °C in Dulbecco’s modified Eagle’s medium (DMEM), supplemented with 10% fetal bovine serum (FBS) and 1% penicillin/streptomycin each.

### Preparation of GFP-HTT-expressing cells

U87MG cells (10^4^ cells/well) were seeded in 12-well plates. LipofectamineTM 3000 transfection reagent (Invitrogen) was used for the transfection of GFP-HTT poly Q74 exon 1 DNA into U87MG cells. Typically, GFP-HTT poly Q74 exon 1 DNA was transfected into target cells for 24 h, and the cells were treated with LPPs.

### Preparation of amyloid beta oligomer

Amyloid β protein (1–42) (BACHEM) was monomerized with 1,1,1,3,3,3-hexafluoro-2-propanol (HFIP) for 3 days to prevent β-sheet structure formation. The solution was dried into a peptide film via a SpeedVac and stored at -80 °C. DMSO was added the day before amyloid beta was added, the mixture was resuspended through water sonication, and serum-free medium was added. The concentration of amyloid beta was 10 µM, and oligomerization was carried out at 4 °C for one day. To verify the composition of Aβ oligomers, Western blot analysis was performed under non-reducing conditions after oligomerization. It revealed distinct bands corresponding to monomers (~ 4.5 kDa), dimers (~ 9 kDa), and higher-order oligomers. SK-N-SH cells (10^3^ cells/well) were seeded in 6-well plates and treated with Aβ oligomers for 24 h.

### Acidic organelle staining assay

U87MG and SK-N-SH cells were incubated with LPPs for 6 h at 37 °C. After incubation, lysosomal pH was analyzed using multiple staining methods. For LysoTracker™ Red DND-99 (Invitrogen), cells were stained with a final concentration of 50 nM for 15 min at 37 °C. LysoSensor™ Yellow/Blue DND-160 (Invitrogen) was applied at a concentration of 2 µM for 3 min under the same conditions to assess lysosomal pH ratiometrically. Additionally, LysoSensor™ Green DND-189 (Invitrogen) was used at a concentration of 2 µM for 20 min to measure lysosomal acidification based on fluorescence intensity. Thereafter, the medium was removed, the cells were washed twice with PBS, and observed via an Evos FL Auto 2 cell imaging system.

### Measurement of lysosomal pH

Cells were seeded into a 96-well plate and incubated with 1 µM LysoSensor™ Yellow/Blue DND-160 (Invitrogen) for 5 min. After incubation, cells were washed once with fresh media. For test wells, the media was replaced with HBSS supplemented with 10% fetal bovine serum (FBS) for 10 min. To generate a standard curve, separate wells were incubated with a calibration buffer (25 mM HEPES, 115 mM KCl, 1.2 mM MgCl, 10 mM glucose, 10 µM nigericin) adjusted to pH 4.5, 5.0, 5.5, 6.0, 6.5, or 7.0, as described previously [66]. Fluorescence intensity was measured using a PerkinElmer EnSpire multimode plate reader with excitation/emission filters set to 329/440 nm and 380/540 nm.

### Cathepsin activity assay

U87MG cells and SK-N-SH cells (10^5^ cells/well) were seeded in 6-well plates. The cells were treated with GFP-HTT Q74 exon 1, BafA1, or Aβ and incubated with LPPs for 6 h. Cathepsin activity was measured using the Cathepsin Activity Fluorometric Assay Kit (BioVision), following the manufacturer’s protocol. Harvested samples were washed with PBS and lysed with cell lysis buffer. The samples were centrifuged for 5 min at 4 °C at 13,000 rpm to remove insoluble material, after which 50 µl of the supernatant was transferred to black 96-well plates, and 50 µl of reaction buffer and 2 µl of substrate were added. The cells were incubated at 37 °C for 1–2 h in the dark. The output was measured via a fluorescence microplate reader. Cathepsin B activity assay kit (Fluorometric) (ab65300, Ex/Em = 400/505 nm); Cathepsin D activity assay kit (Fluorometric) (ab65302, Ex/Em = 328/460 nm); Cathepsin K activity assay kit (Fluorometric) (ab65303, Ex/Em = 400/505 nm); and Cathepsin L activity assay kit (Fluorometric) (ab65306, Ex/Em = 400/505 nm).

### Cell viability assay

Cell viability was measured using the CellTiter 96 Aqueous One Solution Cell Proliferation Assay Kit (Promega), following the manufacturer’s protocol. U87MG cells and SK-N-SH cells (5 × 10^3^ cells/well) were seeded in 96-well plates. The cells were treated with GFP-HTT Q74 exon 1, BafA1, or Aβ and incubated with LPPs for 24 h. Then, 20 µl of CellTiter 96 Aqueous One Solution reagent was added to each well containing samples in 100 µl of culture media. The cells were incubated at 37 °C for 2 h in a humidified atmosphere containing 5% CO_2_. The absorbance output was measured at 490 nm using a microplate reader.

### Cell cytotoxicity assay

Cell cytotoxicity was measured using the CytoTox 96 Non-Radioactive Cytotoxicity Assay Kit (Promega), following the manufacturer’s protocol. U87MG cells and SK-N-SH cells (5 × 10^3^ cells/well) were seeded in 96-well plates. The cells were treated with GFP-HTT Q74 exon 1, BafA1, or Aβ and incubated with LPPs for 24 h. Next, 50 µl aliquots were transferred from the samples to a new 96-well plate. To these wells, 50 µl of CytoTox 96 reagent was added, and the cells were incubated at room temperature for 30 min in the dark. Finally, 50 µl of stop solution was added, and the output absorbance was measured at 490 nm.

### Western blotting

U87MG cells and SK-N-SH cells were lysed with RIPA buffer (150 mM NaCl, 1% Triton X-100, 1% sodium deoxycholate, 0.1% SDS, 50 mM Tris (pH 7.5), and 2 mM EDTA) containing a protease inhibitor cocktail. Equal amounts of protein samples were separated via 10% SDS‒PAGE and transferred to a PVDF membrane. The primary antibodies used in this study were as follows: anti-p62 (1:1000 dilution, Cell Signaling, 5114 S), anti-LC3 (1:1000 dilution, Abcam, ab51520), anti-beta-actin (1:10000 dilution, Sigma‒Aldrich, A1978), anti-GFP (1:1000 dilution, Santa Cruz, sc-9996), anti-amyloid beta (1:1000 dilution, Cell Signaling, 2450T), and anti-LAMP2A (1:1000 dilution, Abcam, ab18528). Bands were detected via ImageQuant 800 (AMERSHAM) and quantified via ImageJ.

### mCherry-GFP-LC3 assay

U87MG cells (10^4^ cells/well) were seeded in 12-well plates. mCherry-GFP-LC3 was transfected into the target cells for 24 h, and the cells were treated with LPPs plus bafilomycin A1 (BafA1) at 200 nM for 6 h. The cells were fixed with 2% formaldehyde solution and permeabilized with 0.5% Triton X-100. To indicate the nucleus, DAPI staining was performed, and mounting was conducted via anti-fade fluorescence mounting medium (Abcam).

### Aggresome detection assay (PROTEOSTAT)

U87MG cells (10^4^ cells/well) were seeded in 12-well plates. The cells were treated with LPPs or 5 µM MG132 for 6 h. To stain aggresomes, a PROTEOSTAT Aggresome Detection Kit (Enzo) was used, following the manufacturer’s protocol. The cells were fixed with 2% formaldehyde solution and permeabilized with a permeabilizing solution. To indicate aggresomes, a dual detection reagent was added for 30 min at room temperature and protected from light.

### Immunocytochemistry

SK-N-SH cells (10^4^ cells/well) were seeded in 12-well plates, and 10 µM Aβ oligomers were applied for 24 h, followed by treatment with LPPs for 6 h. The cells were fixed with 2% formaldehyde solution and permeabilized with 0.5% Triton X-100. Anti- LAMP-2/CD107b (1:200 dilution, Novus Biologicals, NBP2-22217) was used to stain the sections overnight, after which they were stained with an A594-labeled secondary antibody for 1 h.

### Preparation of LPPs with different internal pH liquids

The lysosomal pH-modulating particles contained POPC (850457C, Avanti), DOPC (850375C, Avanti), cholesterol (C3045, Sigma), and NBD-PC (810131P, Avanti) at a molar ratio of 2.04:1:1.3:1.09. The lipids were suspended in chloroform (67-66-3, Sigma) and mixed well in a screwcap tube (1392-150-LP-C, Nacalai USA) according to the molar ratios, and the solvent was removed by evaporation in different vials. After the solvent was completely dried overnight in a fume hood, the dried lamella filmed lipids in five different tubes were hydrated by adding 1 mL of PBS at various pH values (pH 3, 4, 5, 6.5, and 7.4). LPPs were formed by extruding the hydrated lipid films using an Avanti Mini Extruder (610000-1EA, Avanti) through a 200 nm membrane (10417004, Cytiva). After preparing high-concentration LPPs with different pH values, they were diluted with pH 7.4 PBS to a final concentration of 1 mg/mL for cell treatment. The remaining solution that was not incorporated into the liposomes was sufficiently diluted during this process. The pH values of the LPPs were measured using a pH meter. For LPPs containing Cathepsin B (953-CY-010, Bio-Techne), 100 ng of Cathepsin B was added per 100 µL of LPPs during hydration. The preparation procedure and outcome were identical to those used for the synthesis of empty LPPs.

### Statistical analysis

The data are presented as the mean ± standard error (S.E.) from independent biological replicates, unless otherwise specified. Each biological replicate represents an independently prepared cell culture or experiment. Statistical analysis was performed via GraphPad Prism 5. Depending on the experimental design, statistical significance was assessed using either an unpaired two-tailed t-test or one-way analysis of variance (ANOVA) with the Bonferroni correction for comparing individual groups. The protein bands were quantified via ImageJ. Statistical significance was established as *P < 0.05, **P < 0.01, and ***P < 0.001.

## Electronic Supplementary Material

Below is the link to the electronic supplementary material.

Supplementary Material 1

## Data Availability

No datasets were generated or analysed during the current study.
