# Supplementary material for: Lysosomal targeting of liposomes with acidic pH and Cathepsin B induces protein aggregate clearance
Source: Cell Commun Signal. 2025 Jun 19;23:296. doi: 10.1186/s12964-025-02310-z (PMC12180193; doi:10.1186/s12964-025-02310-z)
Supplement: Supplementary file 1 — Supplementary Material 1 [file 12964_2025_2310_MOESM1_ESM.docx]

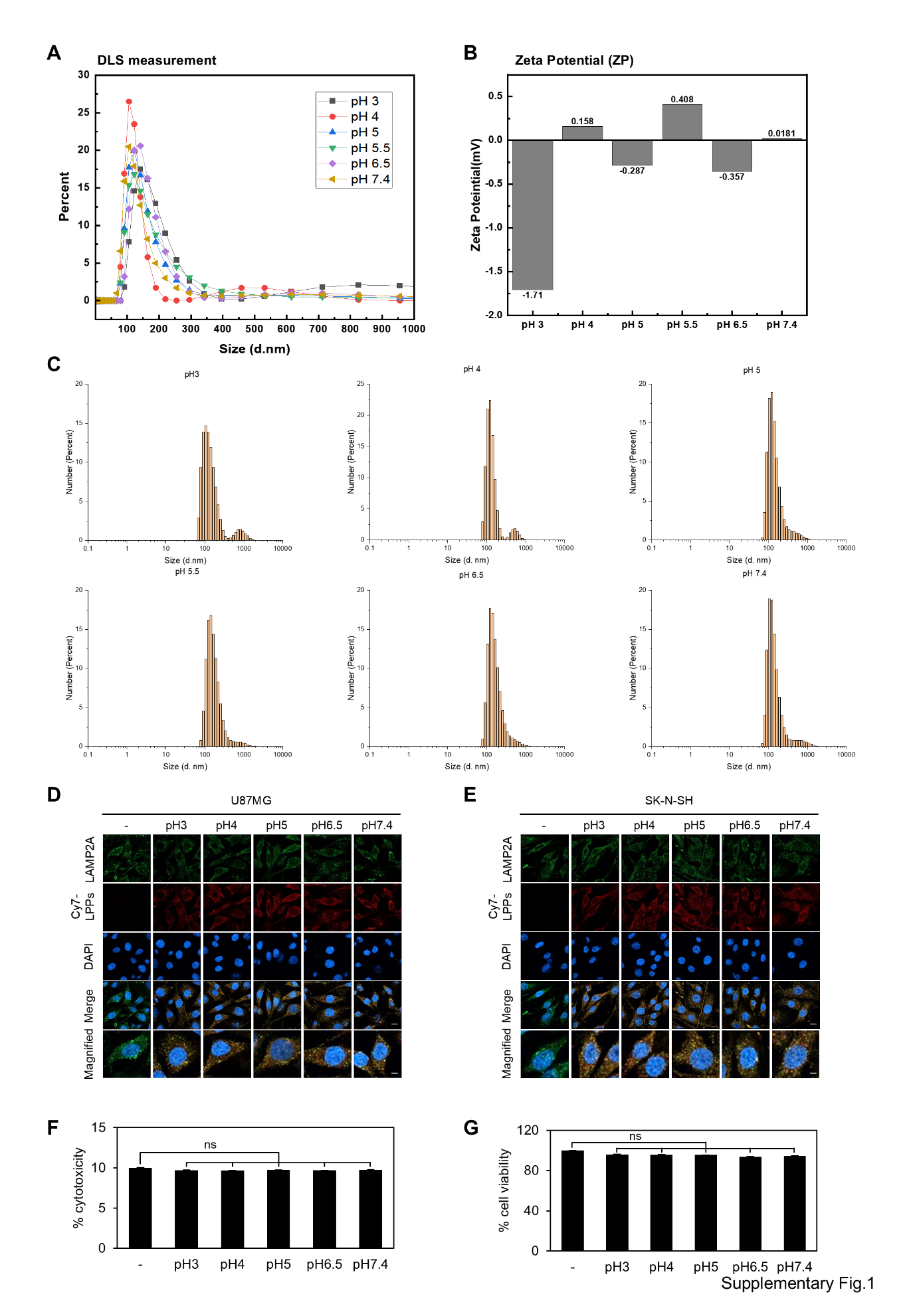


**Supplementary fig 1.**

Fig. S1. Characteristics of LPPs. (A-C) A graph representing the size distribution of dynamic light scattering (DLS), including polydispersity index (PDI) value in PBS (pH 7.4) (D, E) Confocal immunocytochemistry images demonstrating the co-localization of Cy7-labeled LPPs with LAMP2A, a lysosomal membrane marker. Scale bars: 10 μm (overview), 4 μm (magnified). (F, G) Analysis of cytotoxicity and viability 24 hours after treatment with LPPs by LDH (*n* = 5 biological replicates) and MTS (*n* = 8 biological replicates) assay. Data are presented as bar graphs showing mean ± S.E. The p-values were calculated using one-way ANOVA with Bonferroni correction (*, p<0.05, **, p<0.01; ***, p<0.001).


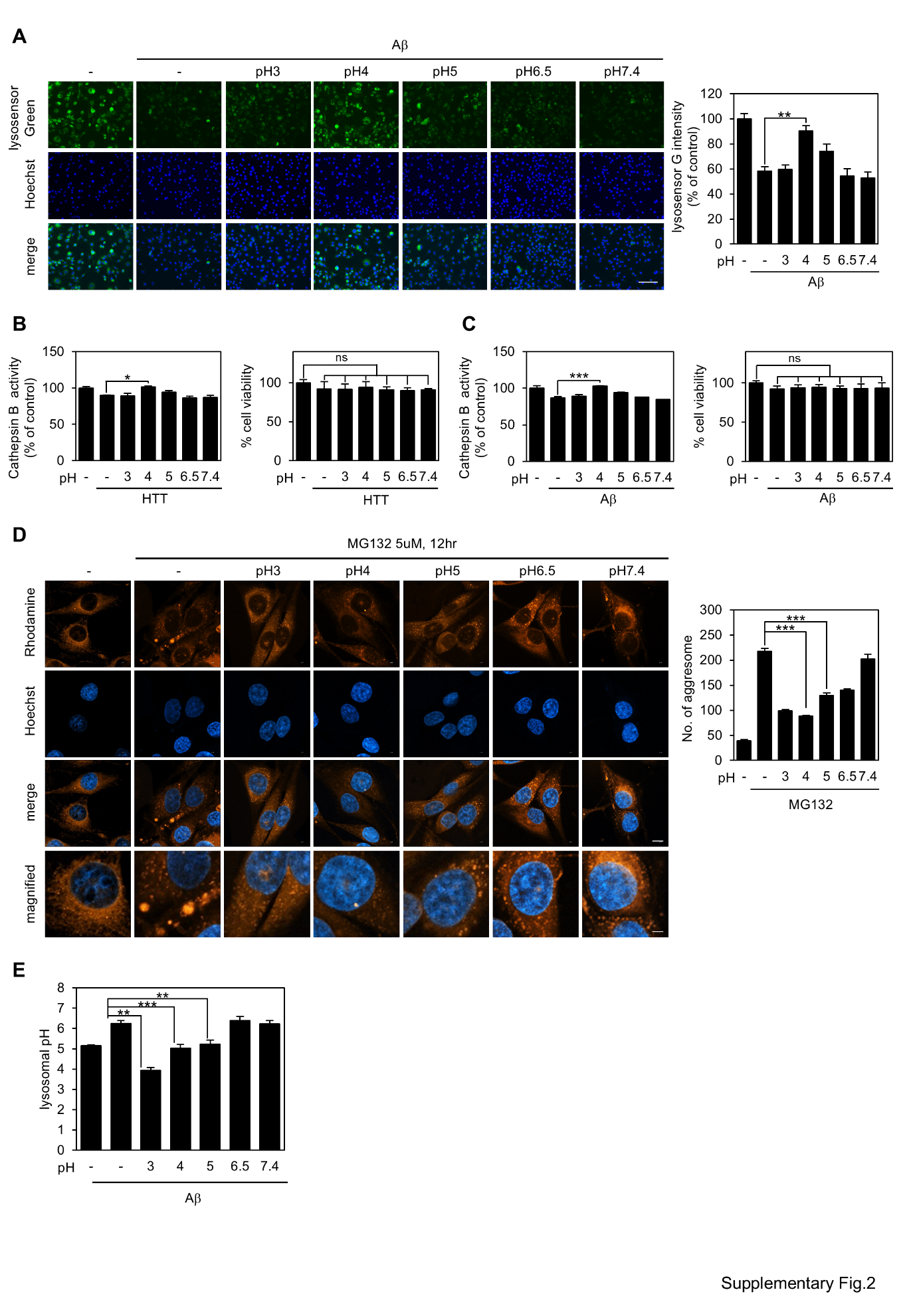


**Supplementary fig 2.**

Fig. S2. LPPs promoted the clearance of protein aggregates by enhancing lysosomal activity. (A) Lysosomal activity analysis using LysoSensor staining in SK-N-SH cells treated with Aβ oligomers and 100 µg/mL LPPs of different pH for 6 hours was quantified per filed (*n* = 5 biological replicates). Scale bar represents 275 μm. (B, C) Cathepsin activity and Cell viability assays were performed under non-lethal conditions in U87MG and SK-N-SH cell lines after overexpression of HTT Q74 exon 1 (0.5µg), and treatment with Aβ oligomers (1µM) for 24 hours, followed by treatment with 100 µg/mL LPPs of different pH for 6 hours. Cathepsin activity was quantified and normalized to the control (*n* = 3 biological replicates). Cell viability was measured by MTS assay. (*n* = 4 biological replicates) (D) U87MG cells were co-treated with 5 µM MG132 and 100 µg/mL LPPs for 12 hours to evaluate the formation and clearance of protein aggregates. Aggresomes were visualized using the PROTEOSTAT® Aggresome Detection Kit following the manufacturer’s guidelines. The number of aggresomes was quantified per cell using ImageJ software (*n* = 5 biological replicates). Scale bars: 10 μm (overview), 3.5 μm (magnified). (E) Lysosomal pH of cells shown in Fig. 3E was quantified using a plate reader following incubation with LysoSensor Yellow/Blue (*n* = 5 biological replicates). Data are presented as bar graphs showing mean ± S.E. The p-values were calculated using one-way ANOVA with Bonferroni correction (*, p<0.05, **, p<0.01; ***, p<0.001).


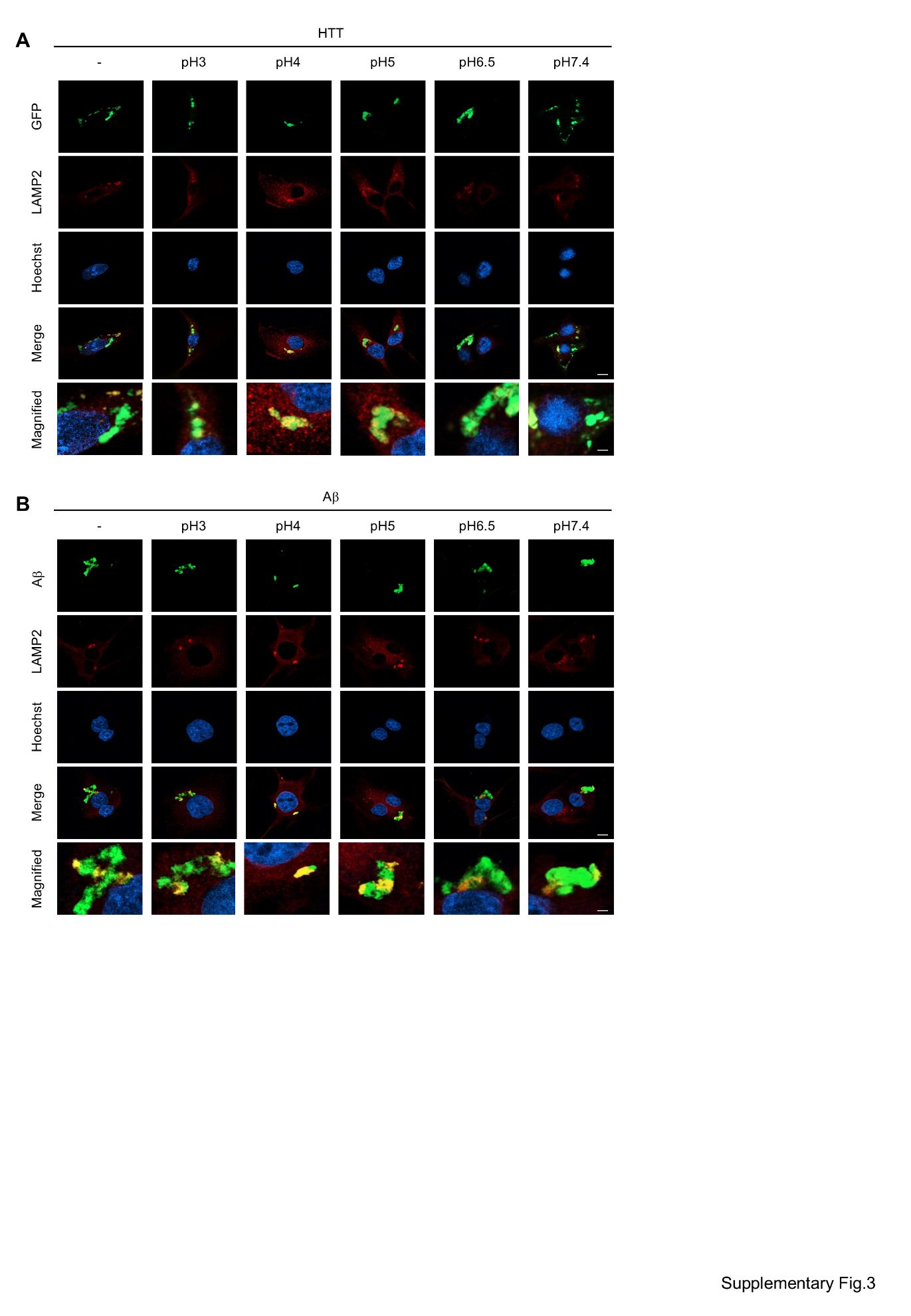


**S****upplementary fig 3.**

Fig. S3. Lysosomal Localization of HTT and Aβ Aggregates Confirmed by Colocalization with LAMP2 after LPP treatment. (A) U87MG cells were transfected with GFP-HTT Q74 exon 1 and treated with various pH LPPs (100 µg/mL) for 72 hours. Scale bars: 10 μm (overview), 2.5 μm (magnified). (B) SK-N-SH cells were treated with 10 µM Aβ oligomers and LPPs of various pH values (100 µg/mL) for 72 hours. Scale bars: 10 μm (overview), 2.5 μm (magnified).


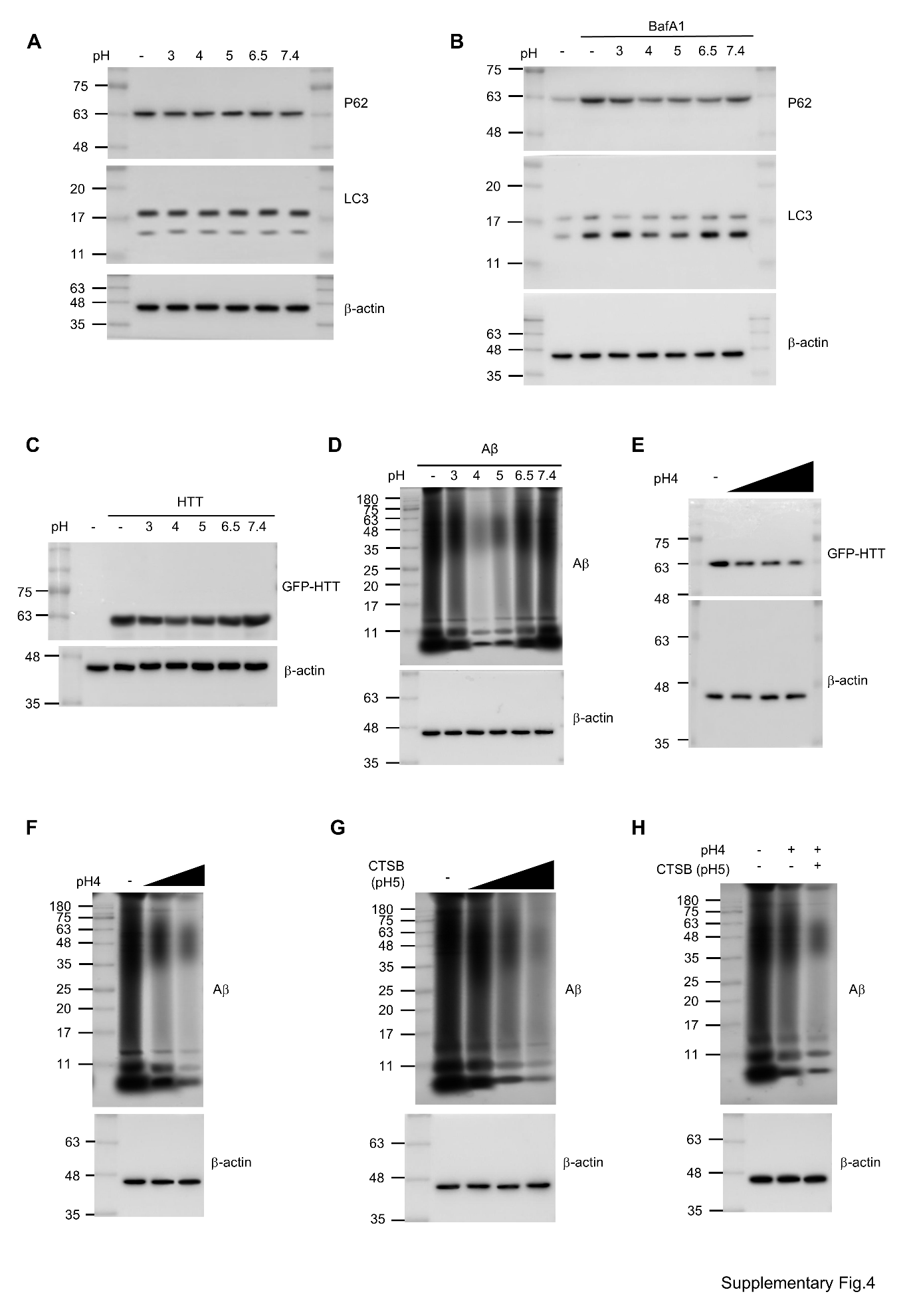


**Supplementary fig 4.**

Fig. S4. Uncropped images of Western blot analyses, including molecular weight markers for reference. (A) Uncropped Western blots presented in Fig. 1I. (B) Uncropped Western blots presented in Fig. 2C. (C) Uncropped Western blots presented in Fig. 4C. (D) Uncropped Western blots presented in Fig. 4D. (E) Uncropped Western blots presented in Fig. 4E. (F) Uncropped Western blots presented in Fig. 4F. (G) Uncropped Western blots presented in Fig. 4G. (H) Uncropped Western blots presented in Fig. 4H. Densitometric quantification of band intensities was performed using ImageJ software, and the quantified values are provided alongside the corresponding images.
